# Relationship between Work-Related Quality of Life and Job Satisfaction in Iranian Occupational Therapists

**DOI:** 10.1155/2021/6692752

**Published:** 2021-09-18

**Authors:** Hamid Reza Rostami, Malahat Akbarfahimi, Amin Ghaffari, Mohammad Kamali, Mehdi Rassafiani

**Affiliations:** ^1^Department of Occupational Therapy, Musculoskeletal Research Center, School of Rehabilitation Sciences, Isfahan University of Medical Sciences, Isfahan, Iran; ^2^Department of Occupational Therapy, School of Rehabilitation Sciences, Rehabilitation Research Center, Iran University of Medical Sciences, Tehran, Iran; ^3^Department of Management, School of Rehabilitation Sciences, Iran University of Medical Sciences, Tehran, Iran; ^4^Occupational Therapy Department, Faculty of Allied Health, Kuwait University, Kuwait

## Abstract

**Objective:**

This study is aimed at exploring the relationship between the work-related quality of life and job satisfaction among Iranian occupational therapists.

**Methods:**

In an analytical-descriptive, cross-sectional study, 322 occupational therapists were recruited through a convenient sampling. Inclusion criteria consisted of age between 22 and 50 years, at least one-year work experience, 24 hours of working per week as an occupational therapist, and membership of Iranian Occupation Therapy Association. After signing the informed consent and completing demographic information, each participant completed the Minnesota Satisfaction Questionnaire- Short Form (MSQ-SF) and work-related quality of life scale.

**Results:**

The highest quality of working life belonged to occupational therapists who were female, married, with an annual salary of more than 40000$ (compared to below 20000 $), and those with work experience between 5 and 12 years. There was a positive and significant relationship between work-related quality of life and job satisfaction. Work-related quality of life scores could significantly predict MSQ-SF scores (*β*1 = 0.54, *p* < .001).

**Conclusions:**

The significantly positive relationship between work-related quality of life and job satisfaction suggests the importance of organizational programs for enhancing factors of work-related quality of life in order to improve job satisfaction and quality of life among Iranian occupational therapists.

## 1. Introduction

Occupational therapy is a client-centered health profession concerned with promoting health and well-being through engaging people in important occupations and activities [1]. Two important issues in any job which are related to overall well-being [2, 3] and employee's productivity and retention [4, 5] are job satisfaction and work-related quality of life. The significant relationship between job satisfaction and work-related quality of life has been reported for different professions [3, 6–8]. To the best of our knowledge, there is no study investigating the relationship between work-related quality of life and job satisfaction in Iranian occupational therapists (OTs). A better understanding of the type of relationship between these two important factors among OTs could provide evidence in designing strategies for better job conditions that can result in better care for clients. Job satisfaction is defined as attitudes and feelings people have about their work. People may have positive attitudes toward their job indicating job satisfaction or negative attitudes that indicate job dissatisfaction [4]. OTs have reported various levels of satisfaction with their job in different countries, cultures, and work settings [9–11]. Factors such as challenging and rewarding work, equitable salary, competitive pay, work settings, adequate staffing, flexible scheduling, job fulfillment, opportunities for personal and professional growth, noticeable progress in the patients' conditions, positive relationships with coworkers, autonomy, a pleasant work environment, reasonable patient-therapist ratio, supervision, and job security have been reported as contributors to job satisfaction among OTs [2, 12, 13].

Work-related quality of life refers to the quality of human experience and condition of life of people as they interact in the employee-employer relationship; in other words, “the favorableness or un-favorableness of a job environment for people” which is more focused on physical and social aspects of the work environment [8, 14]. Better work-related quality of life has been shown to be related to job motivation, job satisfaction, work involvement, life satisfaction, happiness, and decreased self-rated anxiety [3]. According to the literature, factors associated with enhanced work-related quality of life are adequate salary, fair compensation, safe and healthy working environment, opportunities for developing human capacity and career growth, social integration, social relevance of work, and administrative system [8, 14].

The occupational therapy profession was established in Iran in 1971. In 1994, the Iranian Occupational Therapy Association (IROTA) was constituted. Then, IROTA became a full member of the World Federation of Occupational Therapists in 2006. There have been more than 3,000 graduate OTs since the establishment of occupational therapy in Iran [15] that make them very influential in improving quality of care of people with various types of disabilities. As work-related quality of life and job satisfaction influence the health and quality of life of therapists as well as their quality of services, it is essential to examine further these two important variables. Therefore, the purpose of the present study was to explore the relationship between the work-related quality of life and job satisfaction among Iranian OTs.

## 2. Material and Methods

This study was conducted using an analytical-descriptive, cross-sectional method. Participants were recruited from OTs who participated in the 22^nd^ Iranian National Occupational Therapy Congress. Based on convenient sampling, a total number of 322 OTs participated in this study. The inclusion criteria included one year of work experience, working at least 24 hours per week, and membership in IROTA. The inclusion criteria were determined after a consulting session with some professional OTs. During this session, the research team decided to choose participants with different demographic characteristics to ensure nationwide representation. The study protocol was approved by the Iran University of Medical Sciences, ethics committee (approval number: IR.IUMS.REC 1395.95-03-32-28606).

One of the authors responsible for data gathering (A.G.) ensured that the participants met the inclusion criteria through a face-to-face interview. Then, potential participants signed the informed consent and completed the demographic questionnaire, which yielded data on age, gender, years of work experience, educational level, marital status, annual salary, type of employment, work setting, and field of work. In the final step, each participant completed the Minnesota Satisfaction Questionnaire- Short Form (MSQ-SF) and Work-Related Quality of Life Scale (WRQoL) during the congress.

The short version of the MSQ-SF assesses job satisfaction using a 5-point Likert scale ranging from one (extremely dissatisfied) to five (extremely satisfied). It consists of three subscales including intrinsic satisfaction, extrinsic satisfaction, and general satisfaction. As a whole, MSQ-SF contains 20 items, and each item represents a feature in the work environment. The possible scores for MSQ-SF range from 20 to 100 [16]. It is a reliable (0.78) and valid (Cronbach's alpha coefficient: 0.82) questionnaire in Persian language [17, 18].

The WRQoL scale includes 24 items in six subscales evaluating the quality of working life using a 5-point Likert scale. Subscales consist of [1] general well-being (GWB): assesses well-being of an individual from the perspective of overall life satisfaction and mental health problem such as depression and anxiety; [2] home-work interface (HWI): assesses interrelationship and balance between home and work life; [3] job-career satisfaction (JCS): assesses employees level of satisfaction with their job as well as career enhancement and development; [4] control at work (CaW): assesses amount and quality of employees' involvement in decisions that affect work; [5] working conditions (WCs): assesses the fundamental resources and working environment provided for employees to perform their job safely and effectively; and [6] stress at work (SaW): assesses employees' perceptions of exposure to excessive pressure and stress at work. The possible scores for WRQoL questionnaire range from 0 to 100 [19, 20]. It is a reliable (0.95) and valid (Cronbach's alpha coefficient: 0.921) questionnaire in Persian language [21].

Data were analyzed using SPSS Version 16 (SPSS Inc., Chicago, IL, USA). Participants' demographic characteristics were summarized using descriptive statistics. One-way ANOVA and independent *T*-tests were used to compare the WRQoL and MSQ-SF scores between groups (gender, marital status, years of work experience, type of employment, the field of work, annual salary, and educational level). The Pearson correlation coefficient (*r*) was used to determine the relationship of MSQ-SF scores with age, years of work experience, and subscales of WRQoL. Then, a single regression analysis was conducted to assess whether WRQoL can predict job satisfaction; and if it was true, multiple regression analysis was used to predict job satisfaction by subscales of WRQoL. Before performing the hierarchical multiple regression analysis, all presumptions such as multicollinearity by means of Pearson's correlation were checked. In multiple regression analyses, only the factors that were significant in the bivariate correlation analysis were entered into the prediction model. The statistical significant level was set at 0.05.

## 3. Results

Out of 398 distributed questionnaires to OTs who were invited to participate in this study, 322 therapists completed the questionnaire in its entirety (response rate of 80.9%). Majority of the participants were females (53.1%), singles (52.8%), between 22 and 30 years old (77.6%), having between 1 and 4 years of work experience (60.6%), holding bachelor degree (47.2%), working in pediatrics field (50.3%), and employed in private clinics or hospitals (67.7%) (Table 1).

The mean ± standard deviation of MSQ-SF scores was 74.32 ± 10.90 (Table 2). The results of MSQ-SF scores demonstrated that the most job satisfaction belonged to female occupational therapists and those who had between 5 and 12 years of work experience. However, there was not any significant difference in MSQ-SF scores among participants considering their marital status, education levels, fields of work, employment status, and their annual salary (*p* > 0.05).

The mean ± standard deviation of WRQoL questionnaire scores were 82.92 ± 14.17 (Table 2). Female OTs, married therapists, those with an annual salary of more than US$40,000, and those with work experience between 5 and 12 years demonstrated higher level of WRQoL. However, there were not any statistically significant difference in WRQoL scores among participants regarding variables of education levels, fields of work, and employment status (*p* > 0.05).

The correlation analysis showed a significant relationship between MSQ-SF and WRQoL. However, age and years of work experience did not show any significant relationship with MSQ-SF (Table 3). Then, a simple linear regression was performed to test whether WRQoL scores could significantly predict MSQ-SF scores. The results of the regression analysis indicated that the model was significant (*F* (1,320) = 372.92, *p* < 0.001) and the model explained 53.8% of the variance of scores. The results also showed that WRQoL scores can significantly predict MSQ-SF scores in participants of this study (*β*1 = 0.54, *p* < .001). The final predictive model was MSQ − SF score = 27.52 + (0.564^∗^ WRQoL score).

Finally, a multiple regression analysis was performed to predict the MSQ-SF score from different subscales of WRQoL questionnaire. The results demonstrated that all subscales of WRQoL questionnaire except general well-being significantly predicted MSQ-SF score (*F* (7, 314) = 61.48, *p* < 0.001). The model explained 57.8% of the variance of scores (Table 4).

## 4. Discussion

This research is aimed at examining the relationship between job satisfaction and work-related quality of life among Iranian OTs. Based on the results, OTs were moderately satisfied with their job, and they had a moderate work-related quality of life.

Less experienced OTs (1-4 years of work experience) demonstrated the least job satisfaction and work-related quality of life. This finding is in line with other studies that found similar results for less experienced participants [3, 22]. Working independently with clients after graduation from a bachelor degree appears to confront younger OTs with stressful situations. These situations can reduce their work-related quality of life and job satisfaction compared to OTs with more years of work experience [22]. Therefore, providing a formal supervisor or mentor for younger OTs may be a useful strategy for improving new OTs' job satisfaction and work-related quality of life [22].

Female OTs in the present study were more satisfied with the job and had higher work-related quality of life. This finding is in contrast to previous studies that found no significant difference between female and male OTs' levels of job satisfaction [9, 11, 13, 23, 24]. Meade et al. found “opportunities for promotion” and “pay rates” as key factors for job satisfaction/dissatisfaction in males, while for females, teamwork, client contact, respect, and working conditions were the primary factors [11]. More job satisfaction in female OTs in this study could be related to the importance of some factors such as respectful working environments and participation in community situations for women in Iran.

Our results indicated a positive and statistically significant relationship between work-related qualify of life and job satisfaction. Further, the results revealed a statistically significant relationship between the components of work-related quality of life and job satisfaction. It means that improving different aspects of work conditions related to the quality of working life can increase job satisfaction in OTs. Among the components of work-related quality of life, based on regression analysis results, the overall quality of working life and job-career satisfaction were the most important predictors of job satisfaction in OTs, while the general well-being subscale of WRQoL was excluded from the model. Based on the results of previous studies [6] as well as the results of the present study focused on Iranian OTs, quality of working life can predict job satisfaction. It has been reported that the most important aspect of job satisfaction seems to be the work itself [13, 14, 24]. In addition, both work-related quality of life and job satisfaction are related to the retention of employees and lower turnover intention [25]. Furthermore, the work-related quality of life is associated with quality of life [6, 26]. Therefore, by improving work conditions and thereby job satisfaction and work-related quality of life, managers could enhance quality of life among OTs. People with more job satisfaction and better work-related quality of life would have more commitment to the organizations' and thereby contribute to society's overall health and well-being [8, 14] through their service.

There were some limitations worth noting here. As we know, job satisfaction and work-related quality of life and their contributing factors are very culture-based and this put limitations to the generalizability of the results of this study. Convenient sampling and obtaining private information such as salary were other limitations of this study.

## 5. Conclusion

Iranian OTs have moderate work-related quality of life and job satisfaction; with female OTs and those with more than five years of work experience in a better situation. There is a significant and positive relationship between work-related quality of life and job satisfaction. In addition, work-related quality of life can predict job satisfaction. These issues suggest the importance of programs for enhancing factors contributed to better quality of working life in order to improve job satisfaction and quality of life among Iranian OTs.

## Figures and Tables

**Table 1:** The demographic characteristics of participants.

Characteristics	Groups	*n*	%
Work experience	1-4 years	196	60.9
	5-12 years	94	29.2
	13-26 years	32	9.9
Gender	Female	171	53.1
	Male	151	46.9
Marital status	Single	170	52.8
	Married	152	47.2
Education level	Bachelor	152	47.2
	Master	141	43.8
	PhD	29	9
Field of work	Pediatrics	162	50.3
	Adults' mental health	20	6.2
	Adults' physical dysfunction	68	21.1
	All fields	72	22.4
Annual salary	8000-20.000	245	76.1
	>20.000-40.000	66	20.5
	>40.000	11	3.4
Employment status	Government	63	19.6
	Private	218	67.7
	Both	41	12.7

**Table 2:** Descriptive and analytical characteristics of outcome variables in participants.

		Job satisfaction				Work-related quality of life			
Variables	Groups	*M*	SD	*T*/F	*p*	*M*	SD	*T*/F	*p*
Work experience	1-4	73.1	10.5	3.25	0.04	80.9	13.5	5.91	0.00
	5-12	76.5	11.4			86.9	14.9		
	13-26	74.6	10.7			83.2	13.4		
Gender	Female	75.6	10.9	2.25	0.03	84.5	13.6	2.21	0.03
	Male	72.9	10.7			81.1	14.6		
Marriage status	Single	72.2	11.2	-1.89	0.06	81.3	14.2	2.18	0.03
	Marriage	75.5	10.5			84.7	14.0		
Education level	Bachelor	74.4	10.0	1.04	0.36	83.2	13.6	0.35	0.70
	Master	73.7	11.9			82.3	14.7		
	PhD	76.9	9.87			84.5	14.5		
Work field	Pediatrics	74.4	10.9	0.14	0.94	83.1	14.1	1.16	0.33
	Adult mental dis.	75.6	13.7			82.1	15.4		
	Adult physical dis.	74.1	9.75			80.6	13.6		
	All	73.9	11.2			84.9	14.4		
Annual salary	8000-20.000	74.1	10.6	1.05	0.35	82.1	13.6	4.16	0.02
	>20.000-40.000	74.5	12.2			83.94	15.4		
	>40.000	78.9	9.3			94.3	15.5		
Employment status	Government	74.6	9.9	0.14	0.87	79.8	12.5	2.04	0.13
	Private	74.1	11.4			83.5	14.5		
	Both	75.0	10.9			84.8	14.2		

*T*: independent *T*-test; F: one-way ANOVA.

**Table 3:** Correlation analysis of MSQ-SF and WRQoL questionnaires.

	Age	Work experience	General well-being	Home-work interface	Job-career satisfaction	Stress at work	Control at work	Working conditions	Overall quality of working life	WRQoL total
MSQ	0.075	0.074	0.549^∗∗^	0.562^∗∗^	0.695^∗∗^	0.362^∗∗^	0.601^∗∗^	0.648^∗∗^	0.528^∗∗^	0.734^∗∗^

^∗∗^Significant *p* < 0.001.

**Table 4:** Regression analysis to predict job satisfaction from subscales of WRQoL questionnaire.

	Unstandardized coefficients (*β*)	Standardized coefficients (*β*)	95% CI	*p*
General well-being (GWB)	-0.196	-0.075	-0.519-0.127	0.233
Home-work interface (HWI)	0.529	0.130	0.130-0.929	0.010
Job-career satisfaction (JCS)	1.051	0.360	0.714-1.387	<0.001
Control at work (CAW)	0.675	0.113	0.172-1.179	0.009
Working conditions (WCS)	0.516	0.110	0.007-1.024	0.047
Stress at work (SAW)	0.780	0.159	0.147-1.412	0.016
Overall quality of working life	1.895	0.162	0.717-3.073	0.002

Final regression model (*R*^2^ = 0.578, *F* (7, 314) = 61.48, *p* < 0.001).

## Data Availability

Data are available on request through contacting Dr. Malahat Akbarfahimi as the corresponding author (email: akbarfahimi.m@iums.ac.ir).

## References

[1] McHugh Pendleton H., Schultz-Krohn W., Pendleton M. H., Schultz-Krohn W. (2018). *occupational therapy practice framework and the practice of occupational therapy for people with physical disabilities*.

[2] Chen A. H., Jaafar S. N., Noor A. R. (2012). Comparison of job satisfaction among eight health care professions in private (non-government) settings. *Malaysian Journal of Medical Sciences.*.

[3] Zubair M. H., Hussain L. R., Williams K. N., Grannan K. J. (2017). Work-related quality of life of US general surgery residents: is it really so bad?. *Journal of Surgical Education.*.

[4] Mount M., Ilies R., Johnson E. (2006). Relationship of personality traits and counterproductive work behaviors: the mediating effects of job satisfaction. *Personnel Psychology*.

[5] Wegge J., Schmidt K., Parkes C., van Dick R. (2007). “Taking a sickie”: job satisfaction and job involvement as interactive predictors of absenteeism in a public organization. *Journal of Occupational and Organizational Psychology.*.

[6] Kermansaravi F., Navidian A., Navabi Rigi S., Yaghoubinia F. (2015). The relationship between quality of work life and job satisfaction of faculty members in Zahedan University of Medical Sciences. *Global Journal of Health Science.*.

[7] Anbari Z., Abbasinia M., Khadem M., Rahmani A., Asghari M., Ahmad Nezhad I. (2014). Effects of the quality of working life on job satisfaction in an auto parts manufacturing factory. *International Journal of Emergency Mental Health and Human Resilience.*.

[8] Muhamad Noor S., Adli A. M. (2012). Quality Work Life among Factory Workers in Malaysia. *Procedia - Social and Behavioral Sciences.*.

[9] Abu Tariah H. S., Hamed R. T., AlHeresh R. A., Abu-Dahab S. M. (2011). factors influencing job satisfaction among Jordanian occupational therapists: a qualitative study. *Australian Occupational Therapy Journal.*.

[10] Eklund M., Hallberg I. R. (2000). Factors influencing job satisfaction among Swedish occupational therapists in psychiatric care. *Scandinavian Journal of Caring Sciences.*.

[11] Meade I., Ted Brown G., Trevan-Hawke J. (2005). Female and male occupational therapists: a comparison of their job satisfaction level. *Australian Occupational Therapy Journal.*.

[12] Moore K., Cruickshank M., Haas M. (2006). Job satisfaction in occupational therapy: a qualitative investigation in urban Australia. *Australian Occupational Therapy Journal.*.

[13] Mason V. C., Hennigan M. L. (2019). Occupational therapy practitioners' ratings of job satisfaction factors through a lens of social capital. *Occupational Therapy in Health Care.*.

[14] Meena M. L., Dangayach G. S., Bhardwaj A. (2014). Measuring quality of work life among workers in handicraft industries of Jaipur. *International Journal of Industrial and Systems Engineering.*.

[15] Rassafiani M., Sahaf R., Yazdani F. (2018). Occupational therapy in Iran: past, present, and future. *Annals of International Occupational Therapy.*.

[16] Weiss D. J., Dawis R. V., England G. W., Lofquist L. H. (1967). *Manual for the Minnesota Satisfaction Questionnaire*.

[17] Jafar Jalal E., Joolaee S., Hajibabaee F., Bahrani N. (2014). Evaluating the relationship between nurses’ occupational satisfaction and patients’ satisfaction with nursing service. *Iranian Journal of Nursing Research.*.

[18] Hajibabaee F. (2016). Job satisfaction and its effective factors among nurses working in pediatric wards. *Iran Journal of Nursing.*.

[19] Van Laar D., Edwards J. A., Easton S. (2007). The work-related quality of life scale for healthcare workers. *Journal of Advanced N ursing.*.

[20] S A, Va L (2012). *ar DL. User Manual for the Work-Related Quality of Life (WRQoL) Scale: A Measure of Quality of Working Life*.

[21] Mazloumi A., Kazemi Z., Mehrdad R. (2017). Helmi Kohneh Shahri M, Pour Hossein M. Validity and reliability of WRQoL-2 questionnaire for assessment of nurses’ quality of work life. *Health and Safety at Work.*.

[22] Kavanaugh J., Duffy J. A., Lilly J. (2006). The relationship between job satisfaction and demographic variables for healthcare professionals. *Management Research News.*.

[23] Maxim A. J. M., Rice M. S. (2018). Men in occupational therapy: issues, factors and perceptions. *American Journal of Occupational Therapy.*.

[24] Politis I., Tzonichaki I., Gioftsos G. (2015). Job satisfaction of occupational therapists in Greece. *International Journal of Prevention and Treatment.*.

[25] Scanlan J. N., Meredith P., Poulsen A. A. (2013). Enhancing retention of occupational therapists working in mental health: relationships between wellbeing at work and turnover intention. *Australian Occupational Therapy Journal.*.

[26] Narehan H., Hairunnisa M., Norfadzillah R. A., Freziamella L. (2014). The effect of quality of work life (QWL) programs on quality of life (QOL) among employees at multinational companies in Malaysia. *Procedia-Social and Behavioral Sciences.*.

